# Association between Serum Phosphorus Levels and Diabetic Retinopathy: A Cross-Sectional Study

**DOI:** 10.1155/2024/3830246

**Published:** 2024-06-11

**Authors:** Jintao Chen, Chuanfeng Liu, Cunwei Sun, Jia Zeng, Jingwei Chi, Kui Che, Yangang Wang

**Affiliations:** Department of Endocrinology, The Affiliated Hospital of Qingdao University, Qingdao, China

## Abstract

**Background and Aims:**

The aim of this study was to investigate the association between serum phosphate levels and diabetic retinopathy (DR) in patients with type 2 diabetes mellitus (T2DM).

**Methods and Results:**

The study sample consisted of 1657 T2DM patients hospitalized between 2017 and 2019. Patients were categorized into quartiles based on their serum phosphate levels (Q1–Q4). An increasing trend in the prevalence of DR was observed across these quartiles. Subsequently, logistic regression analysis was employed to adjust for potential confounders, such as gender, age, BMI, and duration of diabetes, and to evaluate the odds ratios (ORs) associated with these quartiles. The prevalence of DR showed an increasing trend with elevated serum phosphate levels. Logistic regression further confirmed that serum phosphate levels remain an independent risk factor for DR.

**Conclusion:**

Elevated serum phosphate levels are closely associated with the prevalence of DR in hospitalized T2DM patients. Further studies are needed to establish causality. This trial is registered with chiCTR2000032374.

## 1. Introduction

Type 2 diabetes mellitus (T2DM) represents a chronic metabolic disorder experiencing a swift rise in prevalence across the globe. Among the complications associated with T2DM, diabetic retinopathy (DR) stands out as a prevalent microvascular issue. It has become a primary cause of irreversible vision loss in adults ranging in age from 20 to 74 years [1]. Rigorous regulation of blood pressure and serum sugar levels is essential in the strategic management to hinder the advancement of DR. While laser photocoagulation and the application of vascular endothelial growth factor antibodies have proven to be efficacious therapies, they are part of the broader treatment landscape for this condition [2]. Upon rigorous statistical evaluation of the diabetes control and complications trial (DCCT) study, it has been ascertained that the duration of diabetes and HbA1c levels account for merely 11% of the variability in the risk of developing retinopathy. This finding implies that an overwhelming 89% of the risk variability is attributable to other, as-yet-unidentified, factors [3]. The necessity of researching specific biomarkers related to DR is paramount. By delving into these biomarkers, we can gain deeper insights into the fundamental pathological mechanisms of DR, thereby enabling the development of more targeted and personalized treatment strategies. This not only aids in slowing down the progression of the disease but may also uncover new preventive measures. Therefore, the identification of biomarkers associated with DR and the exploration of innovative retinal intervention methods are key directions for future research, poised to enhance the quality of life for countless patients affected by this condition globally.

In the realm of human physiology, serum phosphate (P), primarily existing in the form of inorganic P, serves as a pivotal electrolyte with multifaceted biological functions. It plays an indispensable role in cellular energy metabolism, particularly in the formation and energy transfer of adenosine triphosphate (ATP). Moreover, serum P is intricately involved in the fine-tuning of enzymatic activities as well as cellular signal transduction pathways. Under normal physiological conditions, the concentration of serum P is typically maintained within a range of 2.5 to 4.5 mg/dL (0.8–1.45 mmol/L) [4]. This homeostatic balance is coregulated by a myriad of factors, including, but not limited to, dietary intake, renal excretion, and the action of parathyroid hormone [5].

In recent years, aberrant levels of serum P have not only been associated with traditional health issues, such as renal dysfunction and skeletal diseases, but have also garnered widespread attention in relation to the onset and progression of various chronic diseases. For instance, some studies have indicated that elevated levels of serum P may be linked to endothelial dysfunction [6], while a high dietary intake of P could potentially induce inflammatory responses [7]. Further research has even suggested that oral administration of high levels of disodium P could induce the onset of diabetic nephropathy [8], and elevated serum P levels are considered a contributing factor to vascular calcification [9]. Additionally, evidence supports the notion that mitochondrial dysfunction and neurodegenerative changes in neurons are related to the deposition of calcium phosphate in mitochondria [10]. A predictive model for diabetic retinopathy was constructed in a retrospective study that included 931 patients with T2DM, which found that gender, insulin use, duration of diabetes mellitus, urinary albumin-to-creatinine ratio, and serum phosphorus were important predictors of the development of DR, but the study did not explore the relationship between serum P and DR in depth [11].

However, it is noteworthy that research exploring the relationship between serum P and DR remains relatively limited. Therefore, the present study aims to delve into the potential association between serum P and DR, thereby filling the existing knowledge gap in this research domain.

## 2. Participants and Materials

### 2.1. Participants

This cross-sectional, real-world observational study was conducted at the Affiliated Hospital of Medicine College, Qingdao University, from 2017 to 2019. A total of 1657 patients diagnosed with T2DM were included. The inclusion criteria encompassed patients aged 18–80 years, diagnosed with T2DM for more than five years, with no gender restrictions. All participants were hospitalized patients. All participants were assessed for retinopathy by a fundus camera (AFC‐330; NIDEX, Kyoto, Japan), slit lamp microscope (3020H; Keeler Ltd., Windsor, UK), and noninvasive optical coherence tomography (5000; Carl Zeiss, Dublin, CA, USA). In accordance with the International Classification of diabetic retinopathy, an ophthalmologist diagnoses the patient based on the results of the examination [12]. Participants displaying signs such as microaneurysms, hemorrhages, hard exudates, venous beading, intraretinal microvascular abnormalities, cotton wool spots, preretinal new vessels, fibrous proliferation, and scars of photocoagulation were identified as those suffering from DR. Nonproliferative diabetic retinopathy (NPDR) included multiple manifestations: microaneurysms and hemorrhages, intraretinal microvascular abnormalities, venous beading, cotton wool spots, and hard exudates. The proliferative diabetic retinopathy group (PDR) is mainly responsible for the formation of neovascularization, which can lead to severe retinal detachment.

The study was approved by the ethics committee of the Affiliated Hospital of Medicine College, Qingdao University (ethics batch number: QYFY WZLL 25748; clinical trial registration number: ChiCTR2000032374).

### 2.2. Data Collection

Anthropometric parameters, including height, weight, and systolic and diastolic blood pressure, were measured. The body mass index (BMI) was calculated using the following formula: BMI = weight (kg)/height^2^ (m^2^). Blood samples were collected after overnight fasting, and the following parameters were assessed: fasting plasma glucose (FPG), fasting insulin, fasting C-peptide, HbA1c, triglyceride (TG), total cholesterol (TC), low-density lipoprotein (LDL), high-density lipoprotein (HDL), serum creatinine (Cr), serum uric acid (UA), serum calcium (Ca^2+^), serum parathyroid hormone (PTH), and serum P. The estimated glomerular filtration rate (eGFR) was calculated according to the Chronic Kidney Disease Epidemiology Collaboration (CKD-EPI) formula. DR was examined using a slit lamp microscope and noninvasive optical coherence tomography (OCT) by an ophthalmologist. Patients were grouped by quartile of serum phosphorus (P): Q1: *P* < 1.08 mmol/L; Q2: 1.08 ≤ *P* < 1.19 mmol/L; Q3: 1.19 ≤ *P* < 1.31 mmol/L; and Q4: *P* ≥ 1.31 mmol/L.

### 2.3. Statistical Methods

Data were analyzed using the SPSS software (Chicago, IL, USA, version 22.0). Continuous variables were expressed as mean ± SD, and categorical variables were expressed as percentages (%). A *P* value <0.05 was considered statistically significant. One-way ANOVA was employed to compare differences between continuous variables, using group Q1 as a reference. The chi-square (*χ*^2^) test was used to compare categorical variables. Logistic regression was utilized to analyze the relationship between serum phosphorus levels and the prevalence of DR.

## 3. Result

### 3.1. Baseline Information

This study encompasses 1657 patients with T2DM, among whom 596 were diagnosed with DR, constituting 35.97% of the total population. All participants were divided into DR and non-DR groups. The duration of diabetes in patients with DR was significantly longer (10.36 vs. 13.61, *P* < 0.05), and the prevalence of hypertension (75% vs. 64.6%, *P* < 0.05) was significantly higher compared to that of the non-DR group. Notably, patients with DR had higher HbA1C (8.31% vs. 8.51%, *P* < 0.05) and serum P levels (1.19 vs. 1.22 mmol/L, *P* < 0.05). Additionally, fasting C-peptide and eGFR were significantly lower in patients with DR (*P* < 0.05). No significant differences were observed between the two groups in terms of gender, age, BMI, and serum lipids (Table 1).

In further analysis, patients were categorized into groups Q1–Q4 according to serum P quartile spacing (1.08, 1.19, and 1.31 mmol/L) (Table 2). Observations revealed a gradually increasing trend in the occurrence of both proliferative and nonproliferative retinal lesions as serum P levels increased (29%, 35.7%, 35.3%, and 36.6% in NPDR; 1.40%, 1.70%, 1.40%, and 3.40% in PDR). Notably, no significant differences in the prevalence of hypertension, fasting glucose, fasting insulin, or HbA1c were seen between the four groups. However, with the increase in serum P, there was a decreasing trend in age (66.87, 63.85, 61.14, 57.71, *P* < 0.05) and duration of diabetes (12.57, 11.7, 11.2, 10.55, *P* < 0.05), which suggests that the increase in the prevalence of DR was not due to an increase in age and duration of diabetes. In groups Q1 to Q4, there was a noticeable decreasing trend in LDL, TC, and TG, while BMI, UA, HDL, and serum Ca^2+^ showed an increasing trend (*P* < 0.05).

### 3.2. Logistic Regression

**Figure 1:**
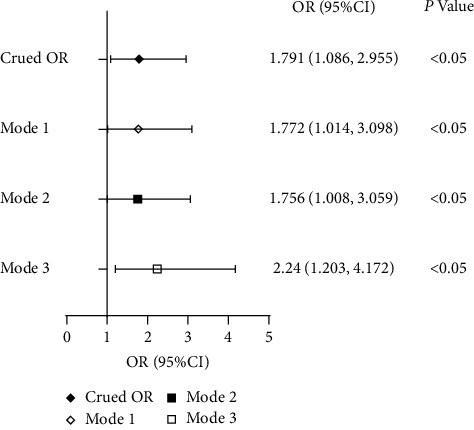
Serum P and DR. Logistic regression analysis unveiled the relationship between serum P and the prevalence of DR: as serum P increased, the prevalence of DR correspondingly escalated. Mode 1: Adjusted for age, gender, duration, BMI, hypertension, drink, and smoke. Mode 2: Adjusted for age, gender, duration, BMI, hypertension, drink, smoke, HbA1c, serum insulin, serum C-peptide, and eGFR. Mode 3: Adjusted for age, gender, duration, BMI, drink, smoke, HbA1c, serum insulin, serum C-peptide, eGFR, TC, TG, HDL, LDL, Mg, Ca^2+^, and PTH. BMI: body mass index, PTH: parathyroid hormone, NPDR: nonproliferative diabetic retinopathy, PDR: proliferative diabetic retinopathy, eGFR: estimated glomerular filtration rate, TG: triglyceride, TC: total cholesterol, LDL: low-density lipoprotein, HDL: high-density lipoprotein, P: phosphorus, Mg: magnesium, Ca^2+^: calcium.

Logistic regression analysis unveiled the relationship between serum P and the prevalence of DR (Figure 1). The analysis indicated that, as serum P increased, the prevalence of DR correspondingly escalated (OR = 1.791, 95% CI (1.086, 2.955), *P* < 0.05). Multiple regression analysis was adjusted sequentially for Mode 1 (gender, age, BMI, duration of diabetes, hypertension, smoking history, and drinking history), Mode 2 (Mode 1 and HbA1c, fasting C-peptide, fasting insulin, and eGFR), and Mode 3 (Mode 2 and Ca^2+^, Mg^2+^, PTH, and serum lipids). The prevalence of DR correspondingly still escalates with serum P increasing (OR = 1.772 in Mode 1, OR = 1.756 in Mode 2, OR = 2.24 in Mode 3, *P* < 0.05).

## 4. Discussion

According to the International Diabetes Federation Diabetes Atlas, the global prevalence of diabetes among individuals aged between 20 and 79 years was estimated to be approximately 10.5%, affecting 540 million people as of the year 2021. This prevalence is projected to escalate to 12.2%, impacting around 780 million individuals by the year 2045 [13]. DR stands as a common microvascular complication of diabetes and is a leading cause of vision loss in the elderly population. Despite its significance, the risk factors associated with DR remain largely elusive [3]. Therefore, the identification of novel risk factors for diabetes and its complications is of paramount importance.

In our comprehensive study, which included a cohort of 1,657 patients with type 2 diabetes, a substantial 35.97% were diagnosed with DR. Our data revealed a direct proportional relationship between elevated levels of serum P and the high prevalence of DR. Compared to patients without DR, those with DR exhibited a longer duration of the disease, along with significantly higher levels of HbA1C and serum P. Although the duration of diabetes was relatively longer in patients with DR than in patients without DR, the duration of diabetes was instead shorter in patients with higher serum phosphorus. This suggests that retinal damage from serum P may occur early in diabetes, independent of the duration of diabetes. Multivariate logistic regression analysis substantiated that the prevalence of DR increased as serum P increased. Our findings are in alignment with the research conducted by Rupal Mehta et al. [14], which investigated a cohort of 1,800 individuals with chronic kidney dysfunction. Their study demonstrated a significant association between serum P levels and existing retinal abnormalities. Interestingly, our study extends these observations by establishing a similar association even among type 2 diabetes patients with normal serum P levels. Mehta and colleagues further corroborated their findings in two prospective cohort studies, namely the Multi-Ethnic Study of Atherosclerosis (MESA) and the Beaver Dam Eye Study (BDES), where they also arrived at similar conclusions [15].

The relationship between serum P levels and diabetic nephropathy has been extensively studied, revealing a commonality of structures, developmental processes, genetic predispositions, and pathogenic mechanisms between the eyes and kidneys [16]. Diabetes serves as a prevalent risk factor for both ocular and renal diseases. In the advanced stages of diabetic nephropathy, elevated levels of serum P are often observed, coinciding with a decline in the eGFR. This phenomenon could partially account for the positive correlation observed between retinal abnormalities and serum P levels. However, it is crucial to note that elevated levels of serum P may not solely be a consequence of renal dysfunction in diabetic nephropathy. Our research indicates that even after adjusting for eGFR, the relationship between serum P and retinal abnormalities remains independently significant. This intriguing observation suggests the possibility of a more intricate and deeper level of association between serum P levels and retinal abnormalities. The choroid is a highly vascularized layer that surrounds and supports the retina, and it plays an important role in the pathogenesis of DR. Kim et al. found a negative correlation between the choroidal vascular index, systemic and ocular profiles of subfoveal choroidal thickness (SFChT), and serum P [17–19]. The above studies suggest that serum P may influence retinopathy by acting on the choroid.

Several plausible mechanisms may underlie the observed positive correlation between serum P levels and the progression of DR. One contributing factor to diabetic retinopathy is the overactivation of the reactive oxygen species (ROS) system. Elevated levels of serum inorganic P have been shown to significantly increase systemic oxidative stress, thereby enhancing the generation of vascular endothelial ROS [20]. Consequently, the overactivation of ROS could potentially serve as a pathway through which elevated serum P contributes to DR. Additionally, research indicates that an increase in extracellular P leads to enhanced intracellular P transport. Studies conducted on isolated mitochondria have demonstrated that when moderate levels of inorganic P increase to supraphysiological intracellular levels, there is a gradual elevation in the production of mitochondrial superoxide [21]. Furthermore, elevated levels of serum P are associated with an increase in Fibroblast Growth Factor 23 (FGF23). Elevated FGF23 levels induce vascular endothelial oxidative stress by activating the NADPH oxidase complex [22]. The FGF23 hormone can directly act on the vascular endothelium, impairing endothelium-dependent vasodilation, especially when the coreceptor alpha-klotho is expressed at lower levels [23–25]. Moreover, evidence supports the notion that mitochondrial dysfunction and neurodegeneration in neurons are related to the deposition of calcium P in mitochondria [10]. DR has been associated with the deposition of calcium and P, and elevated levels of serum P may exacerbate this deposition, potentially serving as another mechanism through which serum P adversely affects the retina.

Angiogenesis plays a pivotal role in the pathogenesis of DR. Fibroblast Growth Factor 21 (FGF21) has been found to be inversely correlated with high dietary P intake in mice [26]. Administration of FGF21 has been shown to inhibit pathological neovascularization in murine models, and mice lacking FGF21 exhibit elevated levels of neovascular formation [27]. A study that stratified DR cases based on severity found that FGF21 levels were positively correlated with the increasing severity of retinal abnormalities in patients with T2DM [28]. Given that the primary source of P in the serum is dietary intake, a reduction in FGF21 levels could potentially be a mechanism through which elevated serum P mediates retinal abnormalities. Furthermore, research utilizing a mouse model lacking klotho expression has indicated that P toxicity accelerates aging, which is associated with the formation of retinal hard exudates [29]. Excessive klotho expression has been observed in states of retinal disease, suggesting that systemic P toxicity could be a contributing factor [30]. We hypothesize that a deficiency in klotho may lead to P accumulation and toxicity, while excessive klotho expression could potentially serve as a compensatory mechanism following elevated serum P levels.

Moreover, diets low in P have been consistently shown to alleviate insulin resistance and improve serum sugar levels, providing a potential avenue for mitigating the effects of DR [31]. On the other hand, elevated P levels are associated with an increased risk of insulin resistance, further underscoring the multifaceted role of P in diabetic complications. In the clinical setting, hemodialysis has been demonstrated to remove between 300 and 1,200 mg of P from the serum per session, offering a therapeutic strategy for managing hyperphosphatemia in patients [32]. Interestingly, hemodialysis has also been found to temporarily enhance insulin sensitivity in individuals with diabetes [2]. This suggests that hemodialysis could serve as a dual-purpose intervention, not only controlling elevated P levels but also improving insulin sensitivity, which could, in turn, have a beneficial impact on DR.

The primary limitation of our study lies in its cross-sectional design, which inherently restricts our ability to draw causal inferences between elevated serum P levels and the onset or progression of retinal abnormalities commonly observed in diabetic patients. This design limitation is particularly noteworthy given the complex interplay of metabolic factors in diabetes and its complications. Moreover, our study sample is exclusively composed of hospitalized patients, which could introduce a degree of selection bias. This limitation not only restricts the generalizability of our findings to a broader diabetic population but also raises questions about the potential influence of hospitalization-related factors, such as acute medical conditions or treatments, on serum P levels. Another point to consider is the potential existence of unobserved confounding variables. While we controlled for known confounders, it is conceivable that other unmeasured factors, such as dietary habits, could have influenced the observed association between serum P levels and retinal abnormalities. For future research, we recommend adopting a longitudinal study design. Such a design would allow for the tracking of serum P levels and retinal health over time, thereby providing a more robust framework for establishing causality. Additionally, expanding the sample size to include a more diverse patient population, both in terms of demographic characteristics and healthcare settings, would enhance the external validity of the study. This would mitigate the selection bias inherent in our current sample and provide a more comprehensive view of the relationship under investigation. Furthermore, coupling clinical data with laboratory studies that delve into the molecular and biological mechanisms linking serum P levels to retinal abnormalities could offer invaluable insights. Such an integrated approach would not only validate the clinical observations but also pave the way for targeted therapeutic interventions.

## 5. Conclusion

Elevated serum P levels are closely associated with the prevalence of DR in hospitalized T2DM patients. Further studies are needed to establish causality.

## Figures and Tables

**Table 1:** Baseline information grouped by DR.

	Non-DR	DR	
Age (year)	62.21 ± 11.605	63.09 ± 10.87	0.128
Gender (female)	44.30%	48.50%	0.1
Duration of diabetes (year)	10.36 ± 7.806	13.61 ± 7.788	<0.05
BMI (kg/m^2^)	26.05 ± 3.596	26.12 ± 3.38	0.683
Systolic blood pressure (mmHg)	139.52 ± 18.955	139.52 ± 18.955	<0.05
Diastolic blood pressure (mmHg)	78.58 ± 11.724	78.5 ± 11.304	0.894
Hypertension (%)	64.6%	75%	<0.05
Smoking (%)	28.70%	27.50%	0.598
Drinking (%)	28.40%	26.40%	0.399
Fasting plasma glucose (mmol/L)	7.38 ± 2.614	7.41 ± 2.575	0.851
HbA1c (%)	8.31 ± 1.872	8.51 ± 1.808	<0.05
Fasting C-peptide (ng/ml)	2.18 ± 1.133	2.02 ± 1.284	<0.05
Fasting insulin (mU/L)	11.84 ± 19.443	13.09 ± 36.948	0.373
Low-density lipoprotein (mmol/L)	2.69 ± 0.905	2.75 ± 1.103	0.173
High-density lipoprotein (mmol/L)	1.21 ± 0.304	1.23 ± 0.321	0.239
Cholesterol (mmol/L)	4.54 ± 1.169	4.63 ± 1.396	0.182
Triglyceride (mmol/L)	1.99 ± 1.989	1.93 ± 1.772	0.523
eGFR (mL/min/1.73 m^2^)	120.47 ± 35.137	114.71 ± 38.439	<0.05
Uric acid (*μ*mol/L)	323.96 ± 89.746	331.2 ± 89.88	0.116
Ca^2+^ (mmol/L)	2.27 ± 0.117	2.26 ± 0.118	0.251
P (mmol/L)	1.19 ± 0.209	1.22 ± 0.196	<0.05
Mg (mmol/L)	0.88 ± 0.081	0.88 ± 0.082	0.324
PTH (ng/L)	34.78 ± 16.35	33.76 ± 22.442	0.3

BMI: body mass index, PTH: parathyroid hormone, eGFR: estimated glomerular filtration rate, P: phosphorus, Mg: magnesium, Ca^2+^: calcium.

**Table 2:** Baseline information grouped by serum P.

	Q1	Q2	Q3	Q4	*P* value
Age (year)	66.87 ± 10.929	63.85 ± 10.316	61.14 ± 10.881	57.71 ± 11.293	<0.05
Gender (female)	35.50%	45.90%	49.10%	53.80%	<0.05
BMI (kg/m^2^)	25.6 ± 3.658	25.92 ± 3.382	26.17 ± 3.626	26.68 ± 3.294	<0.05
Systolic blood pressure (mmHg)	141.77 ± 20.431	140.28 ± 17.411	139.5 ± 19.332	142.38 ± 19.749	0.122
Diastolic blood pressure (mmHg)	78.04 ± 11.554	77.83 ± 10.868	78.21 ± 11.314	80.29 ± 12.448	<0.05
Hypertension (%)	72.6%	69.2%	65.9%	65.3%	0.086
Duration of diabetes (year)	12.57 ± 8.739	11.7 ± 7.961	11.2 ± 7.485	10.55 ± 7.378	<0.05
Smoking (%)	28.70%	27.50%	27.90%	26.40%	0.913
Drinking (%)	30.00%	27.70%	27.60%	27.70%	0.835
Fasting plasma glucose (mmol/L)	7.63 ± 3.013	7.45 ± 2.421	7.32 ± 2.703	7.14 ± 2.097	0.055
HbA1c (%)	8.25 ± 1.928	8.28 ± 1.817	8.5 ± 1.896	8.51 ± 1.737	0.076
Fasting C-peptide (ng/ml)	2.06 ± 1.029	2.12 ± 1.283	2.06 ± 1.052	2.26 ± 1.387	0.075
Fasting insulin (mU/L)	13 ± 39.039	10.92 ± 13.943	12.99 ± 25.936	12.14 ± 21.538	0.66
Uric acid (*μ*mol/L)	308.94 ± 91.647	320.86 ± 82.647	328.04 ± 86.077	350.99 ± 94.019	<0.05
eGFR (mL/min/1.73 m^2^)	115.68 ± 35.876	118.79 ± 35.493	120.7 ± 35.725	118.48 ± 38.794	0.243
Low-density lipoprotein (mmol/L)	2.52 ± 0.905	2.66 ± 0.893	2.77 ± 0.966	2.91 ± 1.121	<0.05
High-density lipoprotein (mmol/L)	1.22 ± 0.327	1.22 ± 0.294	1.25 ± 0.311	1.18 ± 0.303	<0.05
Cholesterol (mmol/L)	4.34 ± 1.163	4.47 ± 1.123	4.67 ± 1.276	4.85 ± 1.399	<0.05
Triglyceride (mmol/L)	1.54 ± 1.164	1.65 ± 1.026	1.92 ± 1.658	2.85 ± 3.004	<0.05
P (mmol/L)	0.9874 ± 0.08691	1.1416 ± 0.03081	1.2518 ± 0.03414	1.4549 ± 0.2178	<0.05
Ca (mmol/L)	2.24 ± 0.119	2.26 ± 0.112	2.28 ± 0.107	2.3 ± 0.121	<0.05
Mg (mmol/L)	0.89 ± 0.083	0.88 ± 0.079	0.87 ± 0.076	0.88 ± 0.087	0.102
PTH (ng/L)	37.66 ± 15.443	33.76 ± 13.129	32.33 ± 12.429	33.8 ± 29.639	<0.05
Non-DR (%)	69.60%	62.60%	63.30%	60.10%	<0.05
NPDR (%)	29.00%	35.70%	35.30%	36.60%	<0.05
PDR (%)	1.40%	1.70%	1.40%	3.40%	<0.05

BMI: body mass index, PTH: parathyroid hormone, NPDR: nonproliferative diabetic retinopathy, PDR: proliferative diabetic retinopathy, eGFR: estimated glomerular filtration rate, P: phosphorus, Mg: magnesium, Ca^2+^: calcium.

## Data Availability

The data used to support the findings of this study are available from the corresponding author upon request.

## Additional Points

*Highlights*. (1) This study aimed to investigate the relationship between serum phosphate levels and diabetic retinopathy in Chinese Type 2 diabetes mellitus patients. (2) Logistic regression analysis was employed to quantify the relationship between elevated serum phosphate levels and the prevalence of DR, confirming its role as an independent risk factor. (3) The study uniquely focuses on a Chinese population, adding specific demographic insights to the existing literature on serum phosphate levels and diabetic retinopathy. *Clinical Trial Registration Number*: chiCTR2000032374.
